# Exploratory analysis of the effectiveness of virtual reality in cardiovascular rehabilitation

**DOI:** 10.1038/s41598-023-50788-9

**Published:** 2024-01-02

**Authors:** Adam Wrzeciono, Błażej Cieślik, Paweł Kiper, Joanna Szczepańska-Gieracha, Robert Gajda

**Affiliations:** 1 Faculty of Physiotherapy, Wroclaw University of Health and Sport Sciences, 51-612 Wroclaw, Poland; 2 Healthcare Innovation Technology Lab, IRCCS San Camillo Hospital, 30126 Venezia, Italy; 3 Gajda-Med District Hospital, 06-100 Pultusk, Poland; 4 Department of Kinesiology and Health Prevention, Jan Dlugosz University, 42-200 Czestochowa, Poland

**Keywords:** Interventional cardiology, Rehabilitation

## Abstract

Virtual reality therapy has been shown to be effective in coping with psychological disorders accompanied by cardiovascular disease. Age appears to be a factor that can affect the effectiveness of psychological therapy in a virtual environment. Therefore, the aim of the study was to explore whether there are age-related differences in the effectiveness of reducing levels of depression and anxiety during a virtual reality psychological intervention implemented for rehabilitation. The study included 25 younger (< 65 years) and 25 older (65 +) patients with cardiovascular disease who participated in virtual reality therapy to cope with anxiety and depression. The Hospital Anxiety and Depression Scale was used to assess anxiety and depressive disorders before and after intervention. Significant reductions in anxiety and depression scores after intervention were observed in both age-matched groups, and no significant differences were found between the younger and older participants. Further evaluation of patient age as a predictor of the effectiveness of psychological intervention in virtual reality did not show a significant effect of age on effectiveness in reducing anxiety and depressive disorders. The results obtained suggest that older patients benefit similarly to younger patients from psychological intervention in a virtual environment. Furthermore, age does not appear to be considered a predictor of effectiveness in reducing the level of anxiety and depression in patients with cardiovascular disease using virtual reality therapy.

## Introduction

Rapid technological development in recent years has resulted in the implementation of modern medical technologies. One of the most explored solutions is virtual reality (VR), with particular attention paid to immersive VR^1^. The benefit of applying immersive VR in clinical settings is that the virtual environment allows the modification of multimodal input stimuli, and thus, patients are more involved in therapy^2^. Basically, VR is categorized into four types: immersive VR, non-immersive VR (referred to as desktop VR and associated with low-cost game consoles), augmented virtual reality (integration of computer-generated data into real-world images), and mixed VR (a blend of real-world elements with virtual entities, controlled either by humans or artificial intelligence)^3^.The immersion effect within immersive VR, achieved by the application of a head-mounted display and headphones, eliminates stimuli from the real environment to create a physical presence in the non-physical (virtual) world. Therefore, patients are able to be distracted from negative sensations during therapy^4^.

Furthermore, the side effects of short-term VR application are generally harmless and rare. When a patient remains in the VR for more than 20 min, the risk of VR sickness (i.e. dizziness, headaches, nausea, and eye strain) can increase^5^. However, caution should be exercised when implementing VR interventions in clinical settings, irrespective of the duration of exposure to the virtual environment. The occurrence of VR sickness is influenced by various factors, including hardware considerations (e.g., display type and mode), content-related aspects (e.g., duration, graphic realism, controllability), and individual predispositions (e.g., susceptibility to motion sickness). Therefore, careful attention should be devoted to monitoring patients' behavior during virtual interventions to promptly identify and address any potential adverse reactions^6^. The effectiveness of virtual reality therapy (VR therapy) has been shown in psychology, especially as a therapeutic intervention to cope with a spectrum of emotional problems, such as depression and anxiety disorders^7,8^. As patients face various problems, psychological disorders are often accompanied by chronic diseases.

In the case of the most serious and dangerous diseases in the world (i.e., cardiovascular disease (CVD))^9^, psychological disorders affect up to one in five patients^10,11^. Depression and anxiety adversely affect the quality of life and daily activity, but more importantly, they are also associated with increased mortality in patients with CVD^12,13^. Many official guidelines for the implementation of comprehensive rehabilitation for cardiovascular disease clearly indicate the need for psychological intervention. Our previous research showed the beneficial effect of the implementation of VR therapy in standard rehabilitation in patients with coronary artery disease and post-stroke patients^14,15^.

The next step in the research on VR therapy is the further development of psychological interventions in the virtual environment and the search for mechanisms responsible for the effectiveness of this technology in both the mental health and functional fitness domains. To explain the mechanisms of action and develop technology in the right direction, it is necessary to explore the factors that may affect the effectiveness of the therapy.

Age appears to be one such factor. Older people are often not convinced of modern technologies and, as a result, are considered "technologically backward". Possible reasons for such a condition include vision disorders, limited physical performance, and an economic state that limits access to modern technology^16–18^. Cognitive impairments related to the aging process^19^ may also be obstacles to proper perception and reactions to therapy in a virtual environment. It would be interesting to assess whether the age of the patient affects the effectiveness of VR therapy.

Therefore, this study aimed to explore whether there are age-related differences in the effectiveness of reducing levels of depression and anxiety during psychological intervention in a virtual environment implemented for rehabilitation. We hypothesize that age could be a predictor of the effectiveness of VR therapy, and older patients may derive less benefit from therapy using modern technologies.

## Methods

### Design

**Figure 1:**
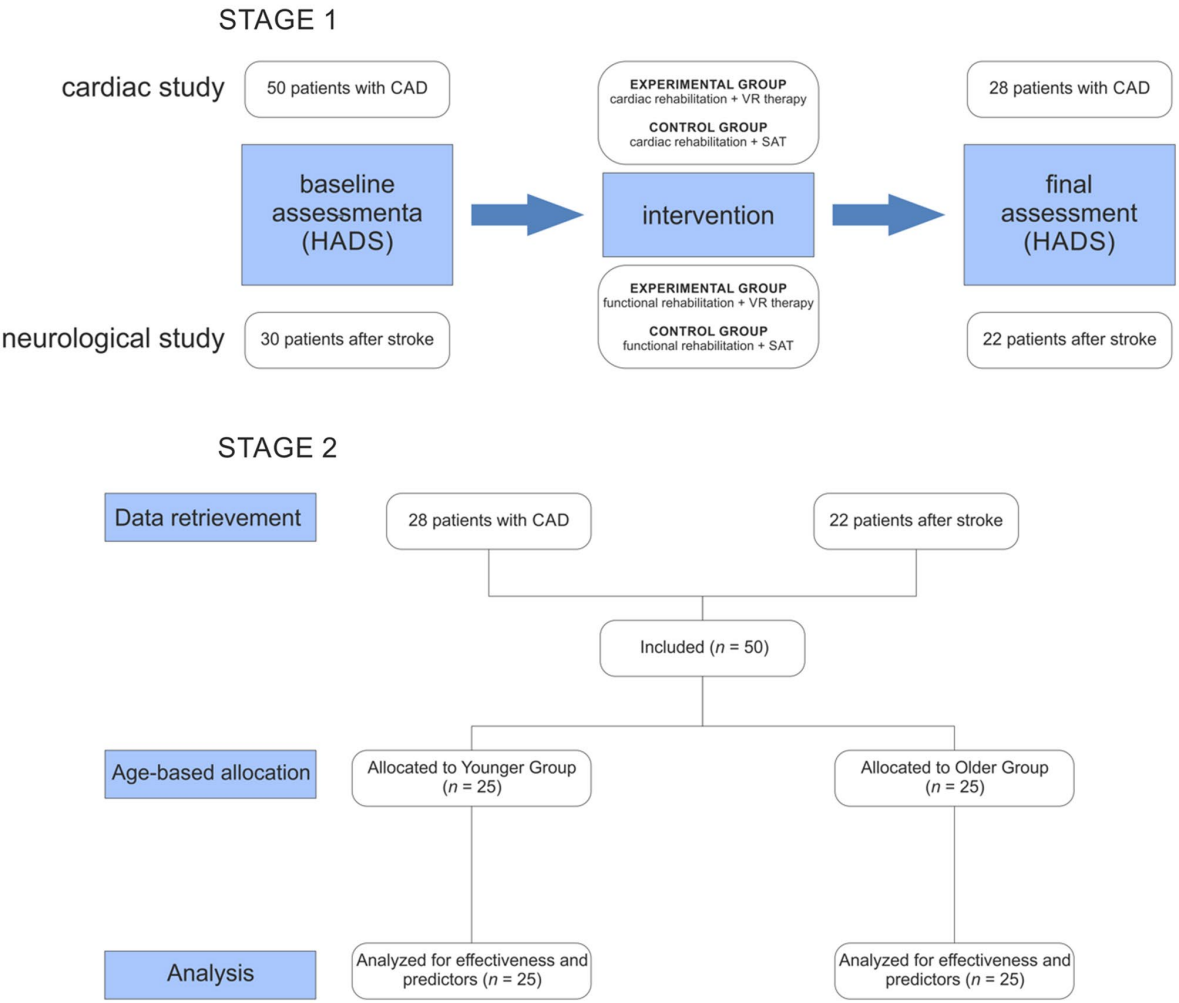
Flow diagram of the study procedure. Stage 1 presents the study flow within the experimental group in both included randomized controlled trials. Stage 2 presents the study flow of the exploratory study. CAD- coronary artery disease, HADS- Hospital Anxiety and Depression Scale, SAT- Schultz Autogenic Training, VR- virtual reality.

We performed an exploratory study nested in two randomized controlled trials (RCT) that evaluated the impact of VR-enhanced rehabilitation in patients with coronary artery disease^14^ and post-stroke patients^15^. Figure 1 shows the detailed study flow. Both studies were approved by the Ethics Committee and registered with ClinicalTrials.gov (NCT04313777 and NCT03830372) and were conducted in accordance with the World Medical Association Declaration of Helsinki. All the participants provided written informed consent for participation.

### Participants

To explore possible age-related differences in the effectiveness of VR therapy, patients assigned to the experimental group from the cohorts of both studies were selected. No restrictions were imposed on the selection process. All participants who completed their scheduled intervention as well as the initial and final assessments were included in the analysis. For the analysis of the measured outcomes, participants were divided by age into two groups: younger (≤ 65 years) and older (> 65 years).

### Intervention

Patients with coronary artery disease participated in standard cardiac rehabilitation, consisting of 40 min of interval training on a cycle ergometer and 40 min of general fitness exercises. The training intensity was individually prescribed based on the calculated heart rate reserve.

Patients after a stroke participated in the standard rehabilitation program, which included 30 min of aerobic training, 30 min of balance exercises, and 60 min of individual rehabilitation following the NDT Bobath concept and proprioceptive neuromuscular facilitation. The treatment aimed to improve the function of both the upper and lower limbs.

**Figure 2:**
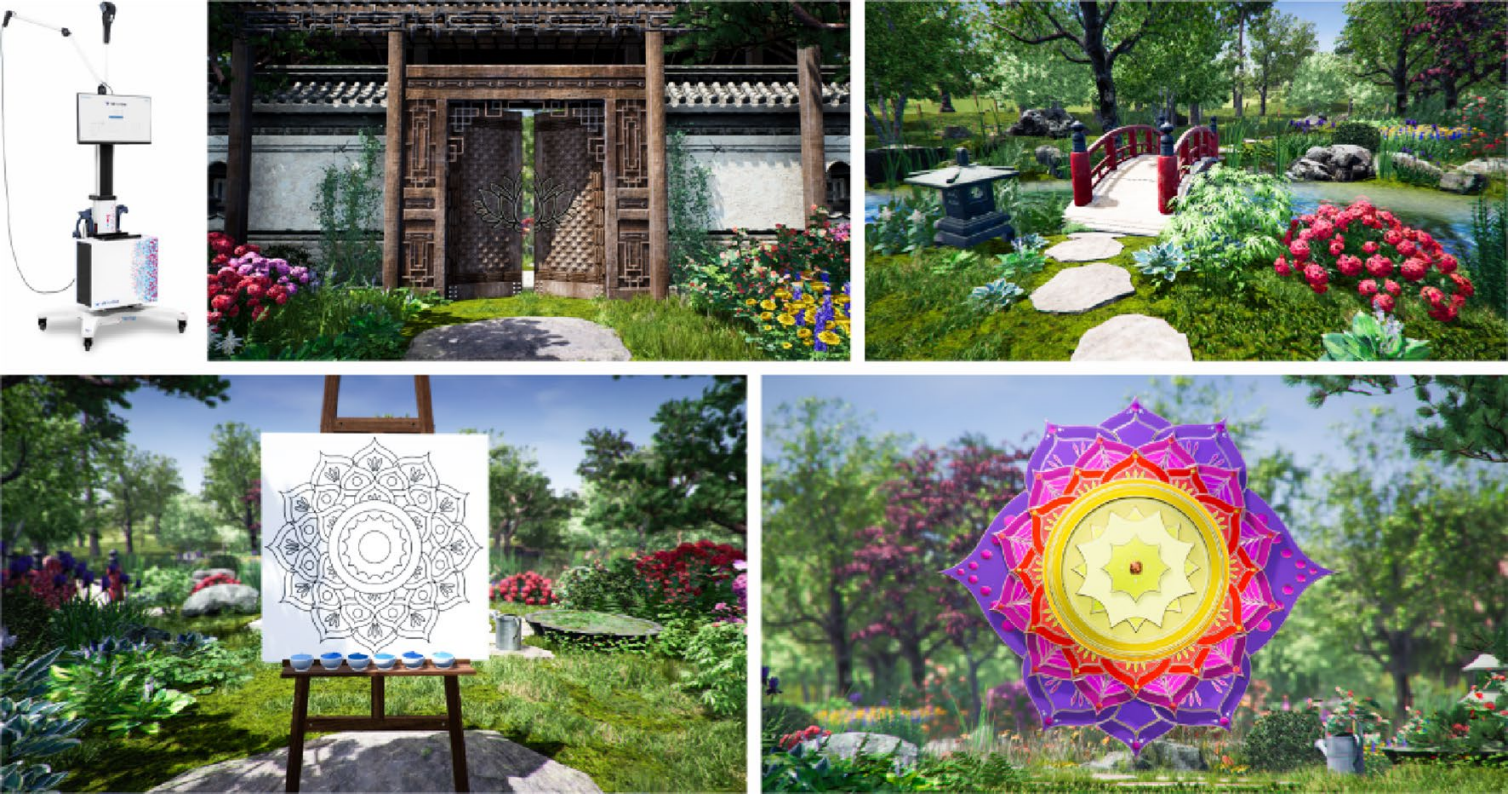
The VR therapy device and environment. sources: Mazurek J, Cieślik B, Wrzeciono A, Gajda R, Szczepańska-Gieracha J. Immersive Virtual Reality Therapy Is Supportive for Orthopedic Rehabilitation among the Elderly: A Randomized Controlled Trial. Journal of Clinical Medicine. 2023;12(24):7681. https://doi.org/10.3390/jcm12247681 and Cieślik B, Juszko K, Kiper P, Szczepańska-Gieracha J. Immersive virtual reality as support for the mental health of elderly women: a randomized controlled trial. Virtual Real. 2023. https://doi.org/10.1007/s10055-023-00797-w.

All participants included in the analysis were treated with the VRTierOne device (Stolgraf, Stanowice, Poland). The VR therapy consisted of 8–10 therapeutic sessions lasting 20 min per session. This therapy was designed to reduce stress and anxiety levels, improve mood, evoke positive associations, and promote patient participation in rehabilitation. The therapy was based on the idea of a Virtual Therapeutic Garden created based on Erickson’s psychotherapy assumptions. Immersion that engages all the senses of the patients and hypnotic communication increases the chances of success of the VR therapy by strengthening patience and perseverance in pursuing a goal. The device consisted of VR goggles (HTC VIVE PRO, New Taipei City, Taiwan) that enabled the display of high-resolution images and dedicated manipulators that transferred patient hand movements to the virtual environment. Visual effects were complemented by the surrounding sounds of the wind, birds, and calm, relaxing music as a background for the messages spoken by the narrator. Figure 2 shows the VR device and the VR therapy environment. A detailed and extended description of this therapy has been provided in previous publications^14,15^.

At the same time, patients in the control groups within both included studies, participated in 8–10 sessions of Schultz Autogenic Training- a psychological component of rehabilitation in control conditions.

### Outcome measures

Outcome evaluations were performed at two time points: before the intervention (T_0_) and after the completion of the VR therapy (T_1_). The primary outcome measure was the Hospital Anxiety and Depression Scale (HADS). The HADS is a standardized tool used to assess levels of depression and anxiety disorders. The questionnaire consists of two subscales related to anxiety (HADS-A) and depression (HADS-D). The scoring for each component ranges from 0 to 21, with cut-off points of 8/21. The higher the score, the greater the anxiety or depression symptoms^20^. Cronbach’s α ranges from 0.78 to 0.93 for HADS-A and from 0.82 to 0.90 for HADS-D; test–retest correlation is r = 0.80^21^.

### Data analysis

The results of the initial (T_0_) and final (T_1_) examinations were obtained, as well as the sociodemographic characteristic of each patient included in the analysis. Data were analyzed using JASP 0.16.1. software (University of Amsterdam, Amsterdam, The Netherlands). Continuous variables are presented as means and standard deviations (SD), and categorical responses are presented as frequencies and percentages. Chi-square tests were used to assess significant associations between categorical variables. The normality of the outcome measures was assessed with the Shapiro–Wilk test. Differences between the two groups were evaluated using independent t-tests. To determine the effectiveness of the intervention, a two-way repeated measures Analysis of Variance (ANOVA) was applied. Multiple linear regression (hierarchical) was used to estimate the possible association between age, gender, and changes in depression and anxiety in patients. A significance level of α < 0.05 was established.

### Ethics approval and consent to participate

The studies from which the results were obtained for analysis were approved by the Institutional Review Board of the University School of Physical Education in Wroclaw. All participants received information regarding the study goals and signed informed consent prior to inclusion.

## Results

### Characteristics of the participants

Data analyzed were obtained from 28 (56%) cardiac rehabilitation patients with coronary artery disease and 22 (44%) neurological rehabilitation patients after stroke. The created age groups (younger and older) were of equal size (n = 25). The characteristics of the younger group were closely matched to those of the older group in all tested domains, except for age, body mass, BMI and employment status (Table 1). However, these differences are the result of age-matched group selection.

**Table 1:** Characteristics of the participants.

Variables	Younger group (*n* = 25)	Older group (*n* = 25)	*p*
Female, *n* (%)	14 (56)	16 (64)	0.56
Age [years], mean (SD)	59.40 (5.56)	72.72 (5.37)	< 0.001
Body mass [kg], mean (SD)	80.86 (13.13)	71.58 (10.98)	0.01
Height [cm], mean (SD)	165.76 (8.80)	164.41 (7.29)	0.62
BMI [kg/m^2^], mean (SD)	29.43 (4.84)	26.41 (3.00)	0.01
Education, *n* (%)			
Primary/vocational	9 (36)	11 (44)	0.13
Secondary	9 (36)	3 (12)	
Higher	7 (28)	11 (44)	
Marital status, *n* (%)			
Single	3 (12)	5 (20)	0.32
Married	15 (60)	12 (48)	
Divorced	2 (8)	0 (0)	
Widowed	5 (20)	8 (32)	
Employment status, *n* (%)			
Employed	8 (32)	1 (4)	0.02
Disability pension	4 (16)	3 (12)	
Retired	10 (40)	20 (80)	
Unemployed	3(12)	1 (4)	
Rehabilitation, *n* (%)			
Cardiac	15 (60)	13 (52)	0.57
Neurological	10 (40)	12 (48)	
HADS, mean (SD)			
T_0_			
Anxiety	6.96 (2.81)	7.76 (4.16)	0.43
Depression	6.28 (3.80)	6.80 (2.99)	0.59
Total	13.24 (6.09)	14.56 (6.39)	0.46
T_1_			
Anxiety	5.84 (3.26)	6.32 (3.65)	0.63
Depression	4.72 (3.39)	5.60 (2.83)	0.32
Total	10.56 (5.66)	11.92 (5.72)	0.40

*SD* standard deviation, *BMI* Body mass index, *HADS* Hospital anxiety and depression scale, *T*_*0*_ Initial assessment, *T*_*1*_ Final assessment.

### Results for the mental state in the studied groups

The results of the primary outcome measures showed a significant decrease in anxiety (*p* = 0.002), depression (*p* < 0.001) and the total score of HADS (*p* < 0.001) between the initial and final measures. However, no significant differences were found between the younger and older group (*p* > 0.05) (Table 2).

**Table 2:** Repeated measures ANOVA results.

	Variables	MS	*F* value	*p* value	η_p_^2^
Time	HADS-A	40.96	10.90	0.002	0.19
	HADS-D	47.61	17.98	< 0.001	0.27
	HADS (total)	176.89	19.23	< 0.001	0.29
Time*Age group	HADS-A	0.64	0.17	0.68	0.004
	HADS-D	0.81	0.31	0.58	0.006
	HADS (total)	0.01	0.001	0.97	< 0.001

*HADS* Hospital Anxiety (A) and Depression (D) Scale, *MS* Mean square, *ƞ*_*p*_^*2*^ Partial eta squared.

### Factors for predicting anxiety and depression level after therapy

In order to examine how much age can explain a statistically significant amount of variance in anxiety level after VR therapy, a three-step hierarchical multiple regression was used. In the first step of the regression model, the initial result of the anxiety assessment was included and found to be an important predictor variable, accounting for 48% of the anxiety variance (*p* < 0.001) (Table 3). In the second step, the age was added to the regression model. Among the variables included in the second model, only the initial result of the anxiety assessment variable revealed a significant predictor. This model accounted for 48% (*p* < 0.001) of the variance in anxiety. In the third step, the interaction effect between the initial result of the anxiety assessment, age, and gender was examined. In the third regression model, no changes in significant predictors were found. The third model may explain about 50% of the anxiety variance (*p* < 0.001) (Table 3).

**Table 3:** Results of regression for anxiety.

Variables	b	SE b	95% CI		t	*p*	F	*p*	R^2^	ΔR^2^
			LL	UL						
Step 1							45.05	< 0.001	0.48	0.48
HADS-A pre	0.68	0.10	0.47	0.88	6.71	< 0.001				
Step 2							22.06	< 0.001	0.48	0.00
HADS-A pre	0.68	0.10	0.47	0.88	6.57	< 0.001				
Age	0.003	0.04	-0.08	0.09	0.06	0.95				
Step 3							15.60	< 0.001	0.50	0.02
HADS-A pre	0.66	0.10	0.45	0.87	6.41	< 0.001				
Age	0.005	0.04	− 0.08	0.09	0.13	0.90				
Gender	0.99	0.73	− 0.47	2.46	1.37	0.18				

*HADS* Hospital Anxiety (A) and Depression (D) Scale, *SE* Standard error, *CI* Confidence intervals, *LL* Lower limit, *UL* Upper limit.

To examine how much age can explain a statistically significant amount of variance in the level of depression after VR therapy, a three-step hierarchical multiple regression was used. In the first step of the regression model, the initial result of the depression assessment was included and found to be an important predictor variable, accounting for 57% of the depression variance (*p* < 0.001) (Table 4). In the second step, the age was added to the regression model. Among the variables included in the second model, only the initial result of the depression assessment variable revealed a significant predictor. This model accounted for 58% (*p* < 0.001) of the depression variance. In the third step, the interaction effect was examined between the initial result of the depression assessment, age and gender. In the third regression model, no changes in significant predictors were found. The third model may explain about 58% of the variance of depression (*p* < 0.001) (Table 4).

**Table 4:** Results of regression for depression.

Variables	b	SE b	95% CI		t	*p*	F	*p*	R^2^	ΔR^2^
			LL	UL						
Step 1							64.43	< 0.001	0.57	0.57
HADS-D pre	0.70	0.09	0.52	0.87	8.03	< 0.001				
Step 2							32.60	< 0.001	0.58	0.01
HADS-D pre	0.69	0.09	0.51	0.86	7.88	< 0.001				
Age	0.03	0.03	− 0.04	0.10	0.95	0.35				
Step 3							21.39	< 0.001	0.58	0.00
HADS-D pre	0.69	0.09	0.51	0.86	7.80	< 0.001				
Age	0.03	0.04	− 0.04	0.10	0.93	0.36				
Gender	− 0.23	0.60	− 1.44	0.98	− 0.38	0.70				

*HADS* Hospital Anxiety (A) and Depression (D) Scale, *SE* Standard error, *CI* Confidence intervals, *LL* lower limit, *UL* Upper limit.

## Discussion

Our previous studies indicated that VR therapy seems to have a beneficial effect on the psychological aspect of health in CVD patients. At the current stage of research, it is important to improve technology and explore the mechanisms that can explain the effectiveness of therapy in the virtual environment. Therefore, this exploratory study evaluated whether there are age-related differences in the effectiveness of reducing depression and anxiety levels during a psychological intervention in a virtual environment implemented for rehabilitation.

The age of the patients can be considered as one of the factors that affect the effectiveness of therapy. The relationship between age and the effectiveness of rehabilitation has been demonstrated. Recovering physical fitness becomes more difficult with increasing age of the patients. Older cardiological^22^ and stroke patients^23^ often achieve a lower functional improvement, or a shorter-term improvement, compared to younger patients. Therefore, our hypothesis assumed that age could be a predictor of the effectiveness of VR therapy, and older patients may benefit less from VR therapy compared to younger patients. The analysis of the results did not confirm this hypothesis. The results obtained suggest that age should not be considered as a predictor of the effectiveness of psychological therapy in virtual reality, and older patients benefit similarly from VR therapy as younger patients.

The first aspect to be considered is the effect of age on the severity of depressive and anxiety symptoms. Our results did not show significant differences between the two age-matched groups, and thus age should not be considered as a factor contributing to the severity of depressive and anxiety symptoms. However, the results of the investigation are not unambiguous. The association between age and psychological state is not a simple one-dimensional linear relationship in the general population^24^, so it is even more difficult to determine whether such a relationship exists in people suffering from various diseases.

O'Neil et al. indicated that older adults experienced less anxiety before rehabilitation compared to younger and middle-aged adults and that there were no significant differences in the severity of depression^25^. However, the difference may be due to the fact that O'Neil et al. examined only cardiac patients. The majority were men (68%), and the literature indicates gender-based differences in the prevalence of anxiety–depressive disorders^26^. For stroke patients, it is difficult to find research investigating age-related differences in psychological disorders; however, psychological disorders are common in stroke survivors^27^.

Studies confirmed the association between psychological disorders and lower post-stroke physical activity and a higher level of disability in stroke survivors^22,23^, as well as a negative prognostic factor at all stages of treatment of cardiac diseases^28,29^. Therefore, another aspect to consider is the effect of age on the effectiveness of reducing levels of depression and anxiety. Our results indicate that participants in the two studied groups had elevated levels of anxiety and depression; however, age does not seem to be considered as a predictor of the effectiveness of psychological therapy. This area is poorly researched; however, there are indications that age does not affect the reduction of the severity of depressive and anxiety symptoms^25^. It should be noted that in this study, rehabilitation did not include a psychological component of therapy.

Attention is also drawn to the fact that older patients are less willing to actively participate in rehabilitation and often do not complete their participation in rehabilitation programs^30,31^. This can be of great importance when assessing the effectiveness of rehabilitation. Therefore, it is necessary to look for possible reasons and consequences for such a phenomenon. One factor may be the psychological state of CVD patients. The coexistence of psychological factors (i.e., depressive-anxiety symptoms) and CVD has been extensively studied^27,32,33^. Furthermore, psychological state appears to affect the effectiveness of rehabilitation^34–36^. As a result, there may be a vicious circle effect, where the disease will affect psychological disorders, which in turn will reduce the effectiveness of rehabilitation, which in turn will result in the progression of the disease.

In conclusion, our results appear to be significant. First, we can assume that the age of the patient will not affect the effectiveness of VR therapy. This is a very significant issue, considering that CVD affects younger and younger people; however, the largest proportion of cases still affect older people^37^. Second, the motivational nature of therapy in a virtual environment can help older patients complete the entire rehabilitation process, and thus may slow the progression of the disease. Third, a versatile therapeutic device holds the potential to integrate personalized therapy seamlessly into comprehensive rehabilitation. By translating the foundational principles of Erickson’s psychotherapy into a virtual environment, this device becomes a viable tool for independent use, eliminating the need for the continuous presence of a qualified psychotherapist. Finally, as there may be other disease-related factors that may modify the effectiveness of VR therapy, this area requires further exploration.

### Limitations

This study has several limitations that warrant discussion. First, a small age differentiation in the younger group. To investigate the effect of age even more precisely on the effectiveness of VR therapy, it would be worthwhile to also study young adults and more middle-aged patients. Second, the relation between the psychological state and the functional state was not investigated. This is a very interesting research area that can help improve the rehabilitation process, as well as extend evidence of a relationship between age and the effectiveness of rehabilitation in CVD.

## Conclusions

Our study suggests that age should not be considered as a predictor of the effectiveness of psychological therapy in the virtual environment in patients with CVD. No significant differences in effectiveness between the two studied groups indicate that older patients benefit from participation in VR therapy in a similar way to younger patients. Due to the limitations indicated, the diversity of patients, and the dynamically changing situation, this poorly researched area requires further, extended research.

## Data Availability

Data are available on reasonable request to the corresponding author.
